# Drivers of Change in a 7300-Year Holocene Diatom Record from the Hemi-Boreal Region of Ontario, Canada

**DOI:** 10.1371/journal.pone.0159937

**Published:** 2016-08-17

**Authors:** Kristen K. Beck, Andrew S. Medeiros, Sarah A. Finkelstein

**Affiliations:** 1 Department of Geography, University of Toronto, Toronto, Canada; 2 Department of Geography, York University, Toronto, Canada; 3 Department of Earth Sciences, University of Toronto, Toronto, Canada; Agharkar Research Institute, INDIA

## Abstract

A Holocene lake sediment record spanning the past 7300 years from Wishart Lake in the Turkey Lakes Watershed in the Hemi-Boreal of central Ontario, Canada, was used to evaluate the potential drivers of long-term change in diatom assemblages at this site. An analysis of diatom assemblages found that benthic and epiphytic taxa dominated the mid-Holocene (7300–4000 cal yr BP), indicating shallow, oligotrophic, circum-neutral conditions, with macrophytes present. A significant shift in diatom assemblages towards more planktonic species (mainly *Cyclotella sensu lato*, but also several species of *Aulacoseira*, and *Tabellaria flocculosa*) occurred ~4000 cal yr BP. This change likely reflects an increase in lake level, coincident with the onset of a more strongly positive moisture balance following the drier climates of the middle Holocene, established by numerous regional paleoclimate records. Pollen-inferred regional changes in vegetation around 4000 yrs BP, including an increase in *Betula* and other mesic taxa, may have also promoted changes in diatom assemblages through watershed processes mediated by the chemistry of runoff. A more recent significant change in limnological conditions is marked by further increases in *Cyclotella sensu lato* beginning in the late 19^th^ century, synchronous with the *Ambrosia* pollen rise and increases in sediment bulk density, signaling regional and local land clearance at the time of Euro-Canadian settlement (1880 AD). In contrast to the mid-Holocene increase in planktonic diatoms, the modern increase in *Cyclotella sensu lato* likely indicates a response to land use and vegetation change, and erosion from the watershed, rather than a further increase in water level. The results from Wishart Lake illustrate the close connection between paleoclimate change, regional vegetation, watershed processes, and diatom assemblages and also provides insight into the controls on abundance of *Cyclotella sensu lato*, a diatom taxonomic group which has shown significant increases and complex dynamics in the post-industrial era in lakes spanning temperate to Arctic regions.

## Introduction

Aquatic ecosystems in the 21^st^ century are threatened by numerous anthropogenic disturbances, such as climate warming, land use change, and atmospheric deposition; interactions between these stressors may lead to complex and unpredictable effects [1, 2]. Short-term observational datasets are rarely sufficient to parse out the effects of particular types of environmental change on species diversity or ecosystem function; therefore, paleolimnological approaches are often used to develop conceptual or quantitative models of ecosystem responses to various stressors [3].

While climate may be an ultimate driver of change in aquatic ecosystems, physico-chemical limnological processes are generally the proximate, mechanistic drivers. For example, climate warming in temperate and Arctic lakes affects ice phenology, lengthens the ice-free season and can produce more stable and deeper thermal stratification, thereby affecting habitat available to aquatic biota [4–6]. These processes have been hypothesized to explain major recent changes in freshwater diatom assemblages in regions with seasonal climates, notably, increases in diatoms in the *Cyclotella sensu lato* group, and often concomitant decreases in tychoplanktonic *Aulacoseira* spp. and in benthic diatom taxa [5].

The post-1850 AD increase in *Cyclotella sensu lato* in particular is widespread in many North American lakes, yet is time transgressive. In circum-arctic lakes, rises in *Cyclotella sensu lato* species are often detected at the end of the 19^th^ century whereas in temperate lakes, these increases have been recorded as late as ~ 1970 AD [7]. While increases in the length of the ice-free season explains the recent rise in *Cyclotella sensu lato* at many high latitude sites [8], changes in water clarity and nutrient flux may also be important in explaining increases in these taxa, particularly in temperate locations closer to agricultural and urbanized areas [6]. Tracking and explaining fluctuations in abundances of *Cyclotella sensu lato* diatoms in the pre-industrial Holocene is necessary to better explain the recent responses of this important group of bio-indicators.

In addition to ice phenology, climate also controls lake water level as evaporative balance shifts with hydroclimatic regime [9]. Water level, particularly in a small lake, is a key factor in determining the types of biota, notably the proportion of planktonic vs benthic algae. Holocene paleoclimate has been shown to be a fundamental control on water level in both large and small lakes [10–13] and diatoms are highly responsive to such changes [14]. Despite Holocene paleoclimates which have remained generally humid in Eastern North America, lake level changes on the order of several meters have been documented at many sites [11, 12, 15–17]. Diatoms have been widely used as proxies for reconstructing lake level both qualitatively using diatom growth habits (planktonic to benthic ratios), and quantitatively using calculation of optimal depths along surface sediment transects or other numerical methods reviewed in ref no. [18].

In addition to ice phenology and water level, climate can affect lake ecosystems through vegetation dynamics. In temperate North America, palynological records show a close relationship between forest succession and Holocene paleoclimate [19–22]. Analysis of diatom assemblages in conjunction with pollen records has shown some synchronous shifts in diatom community structure, possibly in part the result of change in local catchment geochemistry brought on by vegetation change [23–27]. Soil development and vegetation succession affect lake water chemistry over both short [28] and long time-scales [29]. For example, an increase in broad-leaved tree species relative to needle-leaved species can influence lake water chemistry due to differences in leaf litter composition [30]. Further, changes in the density of vegetation in catchments can influence the contribution of snowmelt to lakes [31], which can alter water balances [32], and influence nutrient cycling [33]. As a result, the evolution of aquatic ecosystems is influenced by catchment-mediated processes that affect lake-water chemistry, and these are at least partially influenced by climate [5, 29, 34].

Much of our knowledge of long-term changes in biogeochemistry of lakes (lake ontogeny) relies on the analysis of archives of physical, chemical, and biological indicators preserved in lake sediments. The analysis of indicators that respond both directly and indirectly to climate, such as diatoms (Chromista: Bacillariophyta) and pollen, can then be used as proxies for the prevailing environmental conditions of the past. To evaluate the relationship between forest succession, paleoclimate and diatom assemblages, we present a paleolimnological diatom record for Wishart Lake, in the Turkey Lakes Watershed, which has been a monitoring site in northwestern Ontario since 1980. The record from Wishart Lake is interpreted in the context of available regional palynological and paleoclimate data. These comparisons allow for an evaluation of the potential drivers of aquatic ecosystem change over the Holocene, including regional vegetation change, in a region that has undergone large-scale vegetation reorganization since deglaciation. In this paper we aim to answer the following questions:

1. Which environmental factors influence aquatic ecology in the long-term perspective in North Temperate Zone lakes which may be subject to enhanced rates of change with climate warming?
2. Does landscape change play a role in aquatic ecosystem change in ecotonal regions transitional between temperate and boreal forests?
3. How have the abundances of the important bio-indicator diatoms, *Cyclotella sensu lato*, responded to natural environmental variability prior to significant anthropogenic impact?

## Study Site

**Fig 1:**
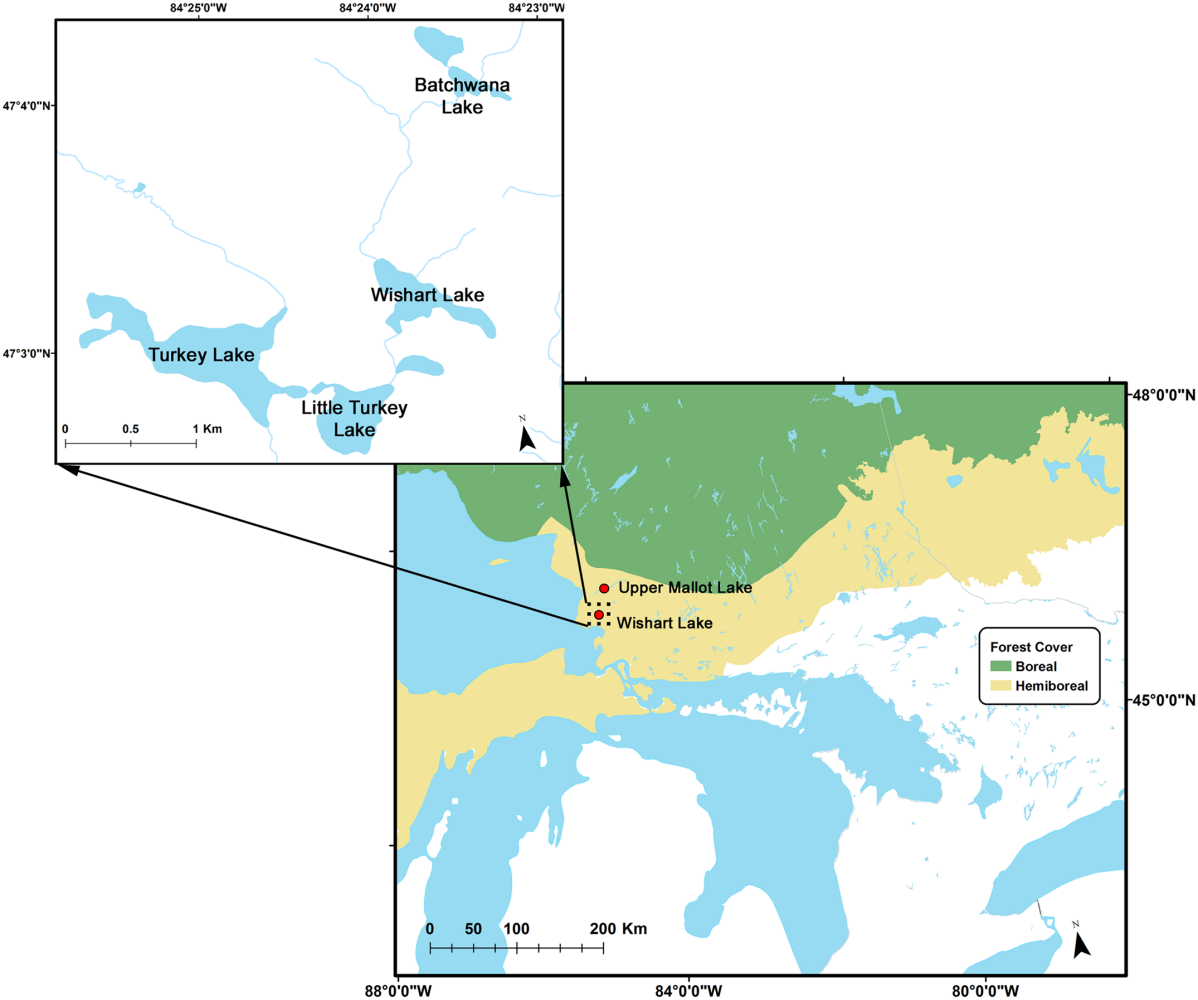
Map of study area. Turkey Lakes Watershed (upper panel) and location of the study lake and nearby Upper Mallot Lake in the context of regional forest zones (lower panel). Data sources: Vegetation data extracted from ref no. [38] and base map layers extracted from the CanVec+ digital cartographic reference product produced by Natural Resources Canada in 2015. Both data sources are licensed under the Open Government License—Canada (http://www.nrcan.gc.ca/earth-sciences/geography/topographic-information/free-data-geogratis/licence/17285). Figures generated using ArcGIS.

Wishart Lake (47.049°N; 84.397°W) is one of four lakes in the Turkey Lakes Watershed (TWL; Fig 1), which encompasses 10.5 km^2^ approximately 20 km inland from Lake Superior in the Province of Ontario, Canada. The bedrock geology of the region consists mainly of Precambrian silicate greenstone (ie., metamorphosed basalt). Till deposits laid down following deglaciation are composed of felsic silt ablation material containing 0–2% calcium carbonate with a thickness on average of 1–2 m [35, 36]. The TLW region was deglaciated between 10–11 ka BP following the Algonquin interstadial [37].

Climate in the TLW is cool and continental. The on-site weather station records a mean annual, July, and January air temperatures of 4.5, 17.8, and -10.7°C respectively (1980–2011). Average annual precipitation (1982–2009) is 1197 mm/yr [39]. The modern day vegetation consists of mixed hardwood forest (Hemi-boreal); the transition to boreal forest is approximately 30 km to the north (Fig 1). Local forest cover is dominated by *Acer saccharum* with mixed hardwoods and conifers present [35]. The lake is polymictic due to summer wind mixing [35, 40]. A bathymetric map of Wishart Lake provided in ref. [35] shows inflow from an adjacent lake at the northwest end, a single basin with maximum depth in its centre, and a shallow channel to the outflow. Monitoring data suggest that the yearly amplitude in water level fluctuation in the TLW does not exceed 1 m, and average water level in a given year deviates from the 20–30 year mean by not more than 1 m [41]. Physical and chemical properties of Wishart Lake are given in Table 1.

**Table 1:** Physical characteristics, median pH, alkalinity and nutrient concentrations for Wishart Lake for 1980–1985. Data from ref no. [35].

Parameter	Value
Elevation (m asl)	390
Surface Area (ha)	19.2
Maximum depth (m)	4.5
Mean depth (m)	2.19
pH	6.7
Alkalinity (μmol L^-1^)	114
DIC (μmol L^-1^)	83.3
DOC (μmol L^-1^)	380
SiO_2_ (μmol L^-1^)	52.6
TP (μmol L^-1^)	0.16
NH_4_^+^-N (μmol L^-1^)	2.64
NO_3_-N (μmol L^-1^)	16.4
TKN (μmol L^-1^)	22.8

Despite its proximity to point sources of sulfate aerosols, and elevated concentration of sulfate in precipitation [36, 42], the pH of Wishart Lake has been stable near ~6.7 over the past 30 years; paleolimnological data for neighboring Batchawana Lake showed no increase in acidophilic diatoms over the past 2–3 centuries [43], suggesting buffering capacity in the catchment. Measured nutrient concentrations in the TLW suggest that the lakes are presently oligotrophic, and apparently limited by phosphorus (Table 1). Owing to protected status and its use as a long-term monitoring site, there has been little disturbance to forest cover within the TLW; however, significant deforestation and industrial activity have taken place in the surrounding region since the end of the 19^th^ century.

## Methods

### Field collection

Permission to access the Turkey Lakes Watershed for lake sediment core sampling was granted to SAF in May 2011 from the Canadian Forest Service (Natural Resources Canada). Lake cores were collected June 2011 at a water depth of 4.5 m, approximately the deepest point in the lake; an anchored coring platform was used to maintain position during core collection. A 7-m lake sediment core sequence (Core WS03) was collected in 1-m sections using a Livingstone piston corer [44]. Woody debris at the base of the core prevented the recovery of sediments below 7 m depth. Because this coring system is not able to collect the uppermost sediments in an undisturbed fashion, a gravity corer [45] was used to collect replicate 26-cm long surface cores (Cores WS02 and WS04) that included the undisturbed sediment-water interface. The two surface cores were taken within <1 m of each other, both contained an undisturbed sediment-water interface, and thus were considered coeval. The Livingstone piston core sequence was collected from the same core hole as the WS02 surface core; the first drive of the Livingstone core sequence was set to commence at the base of the WS02 surface core section. Livingstone core sections were extruded in the field into PVC tubing, and wrapped in plastic wrap and then aluminum foil. The surface cores (WS02 and WS04) were immediately extruded in 1-cm increments.

### Age modelling

An age-depth model was produced from the combination of ^210^Pb activity, the rise in *Ambrosia* pollen, and radiocarbon dating. ^210^Pb dating was performed by drying sediments at 60°C and grinding samples at 1-cm increments. Samples from the surface of the core to depth of 70 cm (WS04/WS03 sequences) were submitted for measurement of ^210^Pb activity by alpha spectroscopy at Flett Research Laboratory (Winnipeg, Manitoba) (Table 2). Five ^14^C dates were selected between the depths of 150–700 cm in the WS03 core sequence; the selected sediment samples were washed with distilled water through a 90-μm mesh. Plant material was selected using a stereomicroscope, and submitted to Beta Analytic Inc. (Miami, USA) for Accelerator Mass Spectrometry (AMS) dating (Table 3). Dates were calibrated using the IntCal09 calibration curve [46] and the program CALIB [47].

**Table 2:** Po-210 activities used to model ages in Wishart Lake core WS04/WS03. Po-210 is assumed to be in secular equilibrium with Pb-210.

Upper Depth (cm)	Lower Depth (cm)	Extrapolated Upper Section Depth (cm)	Core section	Po-210 Total Activity (DPM/g)	Error Po-210 +/- 1 S.D. (DPM/g)	Age at Bottom of Extrapolated Section in Years before 2011 AD (and calendar year) (CRS Model Estimate)
0	1	0	WS04	63.928	2.213	1.3 (2010 AD)
2	3	1.5	WS04	60.892	1.401	4.4 (2007 AD)
5	6	4	WS04	83.724	1.690	10.8 (2000 AD)
9	10	7.5	WS04	83.480	1.648	27.2 (1984 AD)
15	16	12.5	WS04	42.982	1.006	43.8 (1967 AD)
20	21	18	WS04	28.898	0.784	66.3 (1945 AD)
25	26	23	WS04	11.532	0.628	81.4 (1930 AD)
28	29	27	WS03	10.695	0.744	102.4 (1909 AD)
31	32	30	WS03	5.112	0.490	116.0 (1895 AD)
34	35	33	WS03	3.136	0.380	127.3 (1884 AD)
37	38	36	WS03	2.480	0.315	143.3 (1868 AD)
41	42	39.5	WS03	1.942	0.291	168.7 (1840 AD)
45	46	43.5	WS03	1.131	0.231	192.5 (1818 AD)
50	51	48	WS03	0.876	0.247	228.3 (1783 AD)
55	56	53	WS03	0.584	0.323	
70	71	63	WS03	0.500	0.203	

**Table 3:** Wishart Lake Sediment Core (WS03) AMS radiocarbon dates. Dates were calibrated using the INTCAL09 calibration curve and the program Calib v6 [47].

Depth (cm)	Material	Conventional radiocarbon age (^14^C yr BP)	Calibrated age, 2-sigma range (cal yr BP)	∂ ^13^C	Median calibrated age (cal yr BP)	Lab code
142–145	plant (unidentified)	840 ± 30	685–882	-24.7 ‰	750	Beta– 319826
421.5–423.5	plant (unidentified)	3270 ± 30	3407–3571	-23.4 ‰	3500	Beta– 319827
573–574	plant (unidentified)	4450 ± 40	4882–5092	-23.8 ‰	5100	Beta– 321630
687–688	plant (unidentified)	6050 ± 40	6786–7004	-30.1 ‰	6900	Beta– 313894
697–698	plant (unidentified)	6280 ± 30	7164–7264	-26.6 ‰	7210	Beta– 309303

The *Ambrosia* pollen rise was used to confirm the recent chronology and the ^210^Pb dates. The *Ambrosia* pollen rise in Ontario is known to have occurred between 1830 and 1880 AD with precise ages for the *Ambrosia* rise assigned based on local history of forest clearance and disturbance associated with Euro-Canadian settlement [48]. The *Ambrosia* rise in the TLW region is placed at ~1880 AD as this was the time of initial railway development, early mining and settlement in the local area [49, 50].

The *Ambrosia* peak was determined in the Wishart Lake record by processing 32 samples for pollen analysis from the top 65 cm of the record using standard procedures involving acid digestion and sieving [51]. Frequencies of *Ambrosia* and non-*Ambrosia* pollen were identified on a light microscope at 400x magnification for a total of 200 pollen grains. Exotic *Lycopodium* spores were used as markers to determine concentrations of pollen per ml sediment. Two cores collected from Wishart Lake in 1980 AD [52] have data available on the rise in *Ambrosia* pollen and these were used to validate the chronology proposed here.

**Fig 2:**
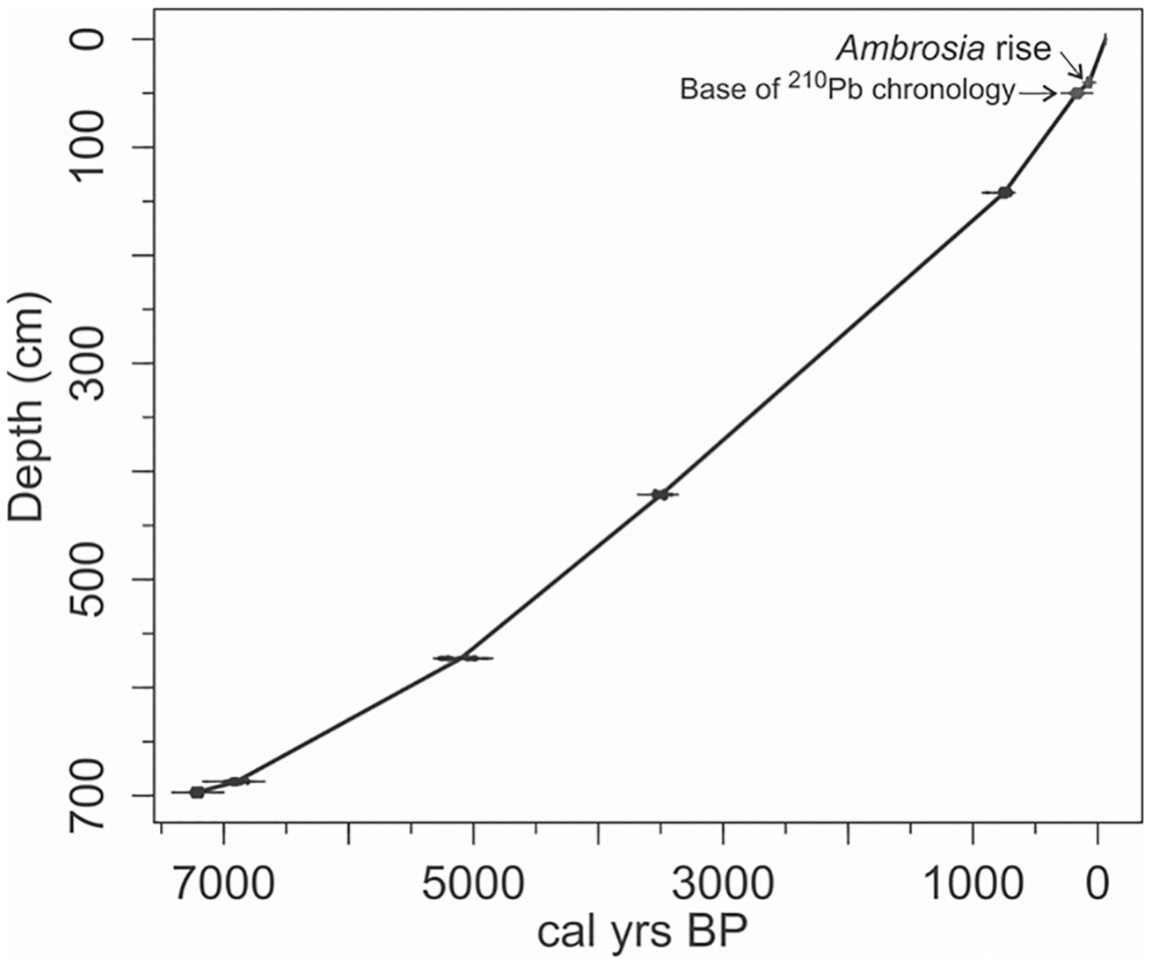
Age-depth model for the Wishart Lake sediment record. The model was developed using a linear regression model fit to calibrated radiocarbon dates, recent chronological data points derived from the *Ambrosia* pollen rise (depth 40 cm) and activity of ^210^Pb (lower boundary used, depth 50 cm). Radiocarbon dates (the five lowermost points) are shown with histograms corresponding to the probability distribution of calibrated dates [53]. Figure generated using *clam* for R [53].

An age-depth model was developed using the date of core collection as an upper constraint (2011 AD), the five radiocarbon dates, the lower-most ^210^Pb date (50 cm depth), and the *Ambrosia* rise (40 cm depth), using the *clam* package for R [53]. The model was based on linear interpolation between dating points, with calculations at 95% confidence ranges and 1000 iterations. Ages were calculated every 1 cm from 0 to 697 cm (Fig 2). Ages for the pre-industrial Holocene are discussed as calibrated calendar years before 1950 AD (cal yr BP); more recent ages are discussed using calendar date (ie. 1950 AD).

### Biological proxies

Point samples from the Wishart Lake core were taken at 10-cm intervals for diatom and chrysophyte cyst and scale analysis, providing an inter-sample resolution of ~100 years. Higher resolution sampling at 1-cm intervals was performed for the upper-most 100 years of the core for an inter-sample resolution of ~4–5 years. Diatoms, chrysophyte cysts and scales, were concentrated using 10% HCl, followed by 30% hydrogen peroxide [54]. Residues of known concentration were mounted with Naphrax^®^. Four hundred and fifty diatom valves were counted per slide using an oil immersion DIC objective on a light microscope at 1000X magnification. Taxa were identified using Antoniades et al. [55], Fallu et al. [56], Krammer and Lange-Bertalot [57], Lavoie et al. [58], and Patrick and Reimer [59]. Diatom taxonomy follows current nomenclature used in Algaebase (http://www.algaebase.org/). Nomenclature for *Cyclotella sensu lato* follows [60]. Loss-on-Ignition (LOI) estimates of inorganic and organic matter in lake sediments were used as a general proxy for sediment provenance, including erosion in the watershed and biological productivity. Standard methods for LOI were applied, with combustion at 550°C and 950°C [61]. LOI_550_ and LOI_950_ were measured every 5 cm, except for the upper 26 cm, where it was measured at 1-cm increments. ANOVA was used to compare the LOI_550_ and LOI_950_ among the diatom-determined zones.

Cluster analysis was used to determine boundaries between diatom assemblage zones in the stratigraphy, with the number of significant zones evaluated by the broken stick model, using the *rioja* package in R [62, 63]. Diatom and pollen assemblages were plotted as stratigraphies using C2 [64]; C2 was also used to ordinate the samples using Detrended Correspondence Analysis (DCA).

To compare our analysis of diatom and chrysophyte indicators with regional paleovegetation and paleoclimate, we extracted pollen and geochronology data for Upper Mallot Lake [20] from the Neotoma database (http://www.neotomadb.org/). Ages were re-modelled for the Upper Mallot Lake pollen record using the top of the core (1993 AD), the three radiocarbon dates included in the database record, and the *Ambrosia* rise (15 cm depth) with the *clam* package for R [53]; pollen assemblages were ordinated using DCA and axis scores were plotted.

All diatom assemblage data, geochronology results and other core data have been deposited in the publicly accessible Neotoma Database (http://apps.neotomadb.org/Explorer/?datasetid=19788). All slides have been permanently archived by the lead author.

## Results

### Age modelling

The activity of ^210^Pb decays regularly with depth, with the exception of lower than expected activity in the upper 5 cm of the core (Table 2), perhaps related to dilution of ^210^Pb or sediment mixing. A constant rate of supply model was used to infer ages from ^210^Pb activities [65]. Background ^210^Pb activity (0.008 Bq g^-1^) is reached at ~56 cm. Because of irregular ^210^Pb activity in the upper 5–6 cm of the core, we interpret that portion of the chronology with caution (2000–2011 AD). Five AMS radiocarbon dates were obtained (Table 3). The section of the core dated by radiocarbon has a similar rate of sediment accumulation to the rates determined using ^210^Pb dates and the *Ambrosia* rise (Fig 2).

To evaluate the proposed age model and the ^210^Pb dates, the *Ambrosia* pollen rise was identified at depth 40 cm and based on local historical records, took place approximately 1880 AD. According to the ^210^Pb age model, this depth corresponds to the year 1860 AD, suggesting an error on the ^210^Pb chronology of ~ 20 yrs. As a further test of the ^210^Pb dates, we also considered *Ambrosia* pollen counts from two Wishart Lake cores taken in 1980 AD, Core 86 and Core 87 described by Harper [52]. In core 86, the *Ambrosia* rise is noted at 24 cm and in core 87, at 29 cm [52]. By taking the difference between the findings of Harper [52] and this study in terms of depth of the *Ambrosia* rise and year of core collection, we assign the year of 1980 AD to the depth of 13 cm in the Wishart Lake core used in the present study. This is based on comparison of the mean depth for the *Ambrosia* rise in the 1982 cores (27 cm) with the depth of the *Ambrosia* rise in the 2011 core (40 cm). The difference, 13 cm, is the depth of sediment accumulated since 1980, and thus the depth of 13 cm in the WS03 core would date to approximately 1980 based on the data of Harper. The ^210^Pb age model proposed here gives a depth of 13 cm a date of approximately 1975 AD. These comparisons suggest errors on the ^210^Pb age model of 5–20 years.

### Diatom biostratigraphy

**Fig 3:**
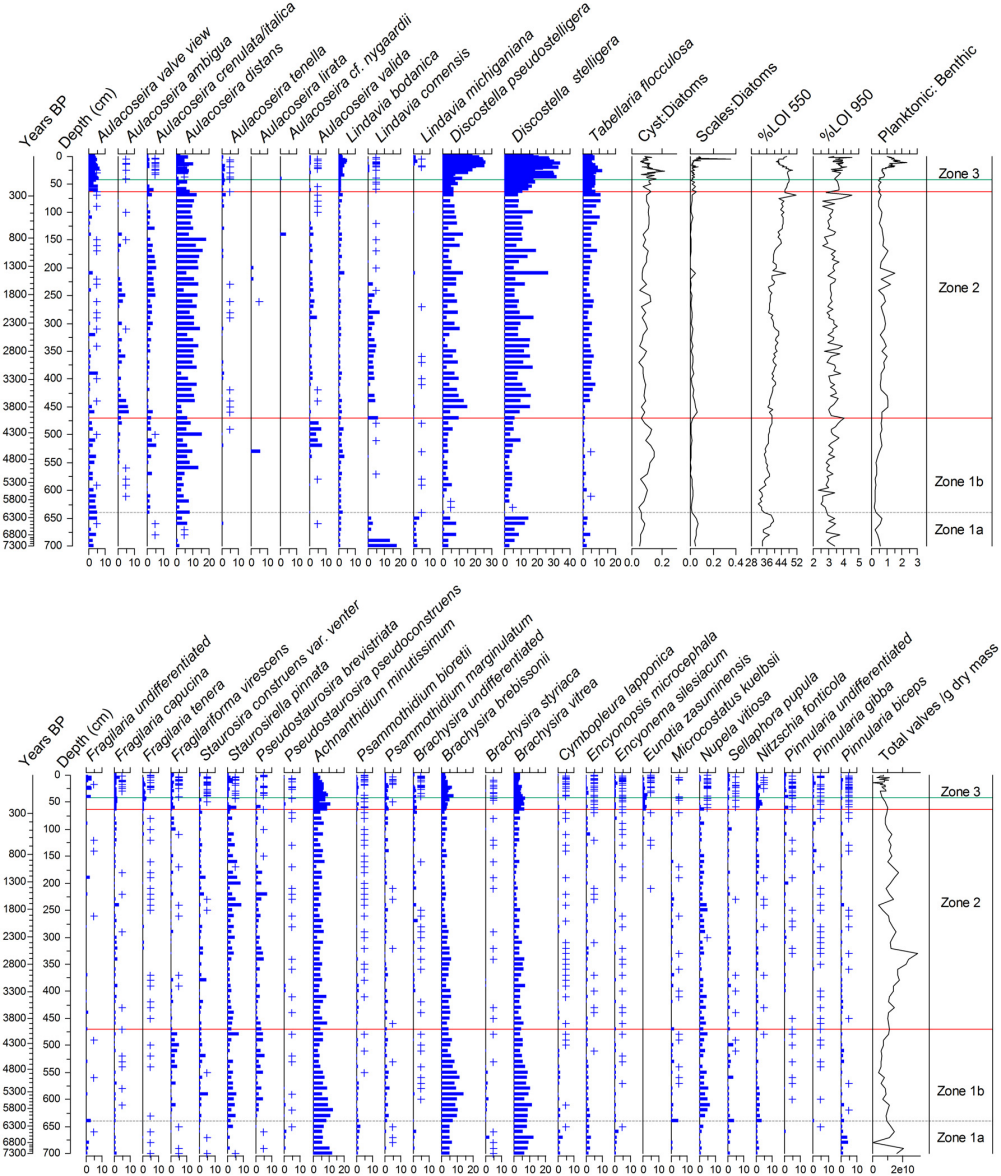
Holocene diatom stratigraphy for Wishart Lake. Diatom species are displayed in percent abundance. Only taxa appearing at >2.5% abundance in any one sample are plotted out of a total of 280 taxa recorded. Sample points where rare taxa appear at <0.5% abundance are displayed by the + symbol. Three significant zones were delineated using cluster analysis (solid red lines, with the dashed grey line separating Zone 1 into two sub-zones). The solid green line in the upper zone indicates the *Ambrosia* rise. The age axis is in calibrated, calendar years BP where year -61 is the time of core collection (2011 AD). Full diatom counts are available at neotomadb.org (Dataset ID = 19788; http://apps.neotomadb.org/Explorer/?datasetid=19788). Diagram plotted using C2 [64].

Approximately 280 diatom taxa were identified from the last ~7300 years in 105 samples. The record is dominated by (tycho)planktonic taxa including (in order of decreasing abundance) *Discostella stelligera* Houk and Klee, *Discostella pseudostelligera* Houk and Klee, *Aulacoseira distans* Simonsen, *Lindavia comensis* Nakov et al 2015, *Tabellaria flocculosa* Kützing, and the benthic taxa *Brachysira brebissonii* Ross in Hartley, *Achnanthidium minutissimum* Czarnecki *B*. *vitrea* Ross in Hartley, *Staurosirella pinnata* Williams and Round, *Pseudostaurosira brevistriata* Williams and Round, and *Nupela vitiosa* Siver and Hamilton (Fig 3). *Discostella stelligera* and *D*. *pseudostelligera* are present through the record generally at abundances between 10–20%. Recent sediments of Wishart Lake contain even higher percentages of *D*. *stelligera* (up to 35%) and *D*. *pseudostelligera* (up to 25%). While it is somewhat surprising to record high abundances of euplanktonic diatoms in a lake with maximum depth of < 5 m, comparable abundances have been recorded for these taxa occasionally in other shallow lakes in the Great Lakes region. For example, Laird et al 2011 [66] report *D*. *stelligera* at abundances of ~10–25% in selected Ontario lakes with depths < 5m to the northwest of the study site; further, Yang and Duthie (1995) [67] in a survey of surface sediments in East Lake and coastal Lake Ontario, report depth optima for *D*. *stelligera* of 1.7 m, *L*. *comesis* of 4. 9 m and *L*. *bodanica* of 3.2 m. While these taxa are clearly planktonic in habit and most often dominant in lakes with depths exceeding 8–10 m [16], these taxa can occasionally be found in shallower lakes, where ecological and limnological conditions permit. Three statistically significant zones were identified in the Wishart Lake diatom record. Zone 1 was split into two subzones (Zone 1a and Zone 1b). Subzones were chosen to facilitate discussion of other shifts in diatom assemblages, and are also supported by nodes in the dendrogram just below the point of significance.

### Zone 1: 7300–4010 cal yr BP (700–470 cm)

**Fig 4:**
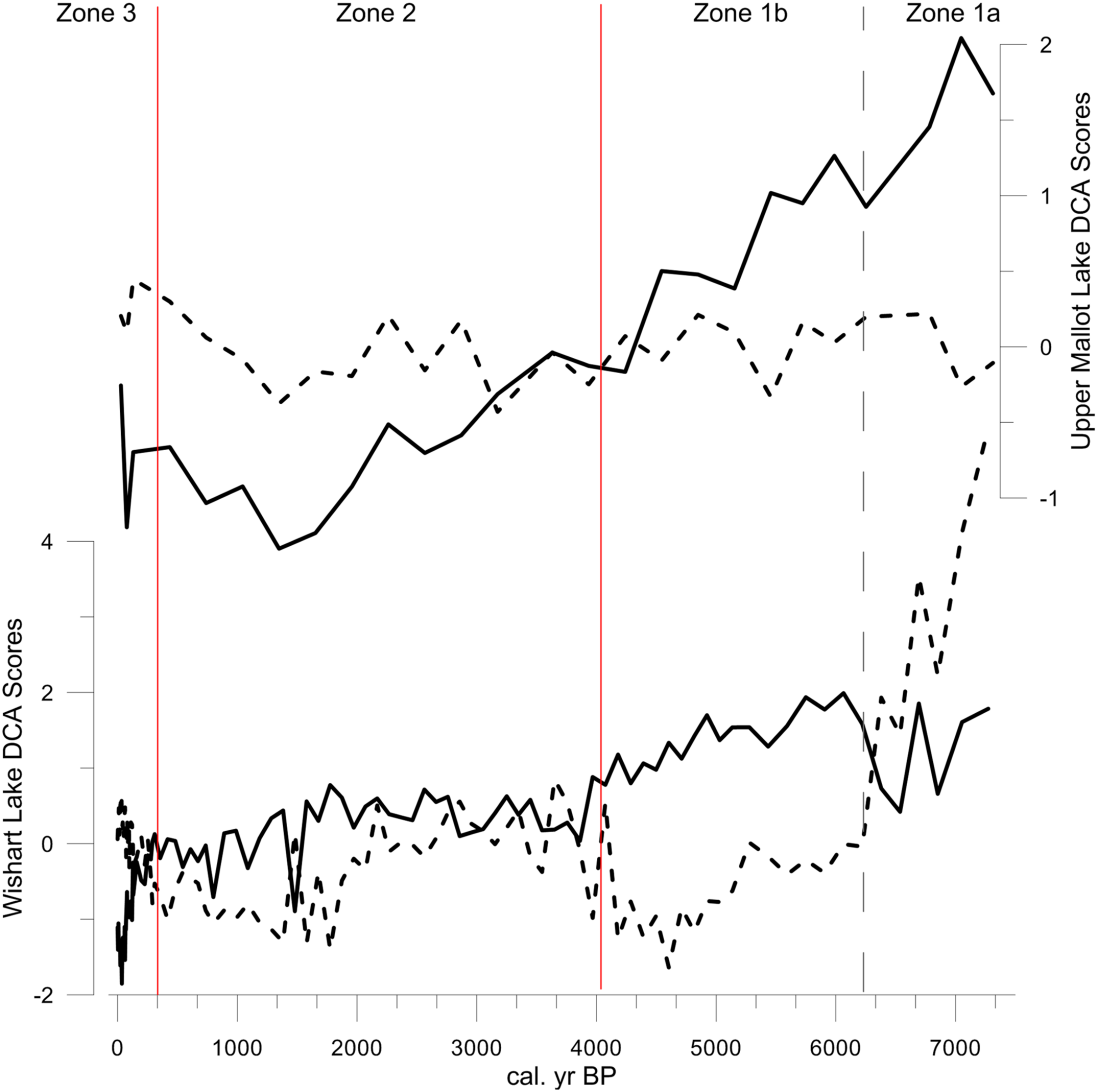
Ordination results for Wishart Lake diatoms and Upper Mallot Lake pollen assemblages. Detrended Correspondence Analysis axis 1 (solid lines) and axis 2 (dashed lines) for Wishart Lake diatom record (lower panel) and for the past ~7200 cal. yr BP Upper Mallot Lake pollen record (upper panel; raw pollen data from ref. [20] and publicly archived at neotomadb.org). Red lines delineate Wishart Lake diatom zones from cluster analysis; dashed grey line indicates the subdivision of Zone 1. Analysis and diagram generated using the *rioja* package for R [62].

Zone 1a (7300–6160 cal yr BP; 700–640 cm) is dominated first by *Lindavia comensis*, then *Discostella stelligera* and *D*. *pseudostelligera*; *Tabellaria flocculosa* and selected benthic taxa, particularly *Achnanthidium minutissimum*, *Brachysira brebissonii*, and *B*. *vitrea*, are also common. The cyst:diatom ratio is low in this zone in comparison to others, while the scale:diatom ratio is higher (Fig 3). The shift to Zone 1b (6160–4010 cal. years BP; 640–470 cm) is characterized by decreasing DCA Axis 1 and Axis 2 scores (Fig 4), an increase in *Aulacoseira distans* from 10 to 25%, an increase in *Pseudostaurosira brevistriata*, *Staurosirella pinnata* and other small benthic fragilarioids from <5 to 10–15% abundance, and an increase in epiphytic taxa such as *Nitzschia fonticola* Grunow in van Huerck, *Nupela vitiosa* and *Pinnularia biceps* Gregory. *Cyclotella sensu lato* spp. decline, along with *T*. *flocculosa*. The cyst:diatom ratio increases in subzone 1b, while the scale to diatom ratio declines (Fig 3).

### Zone 2: 4010–250 cal yr BP (470–63 cm)

The onset of Zone 2 is characterized by marked increases in *Lindavia comensis*, *Discostella pseudostelligera*, *D*. *stelligera* and *Tabellaria flocculosa*. *Aulacoseira* spp. that are dominated by *A*. *distans* remain stable at ~15% of the assemblage. *Fragilaria sensu lato* spp. are somewhat lower in abundance compared to Zone 1b. Cyst:diatom ratios remain constant throughout the zone at ~10% as well as scales:diatoms at ~5%. Both ratios are generally lower in this zone compared to the other zones in the record (Fig 3). DCA axis 1 and 2 scores stabilize with the onset of Zone 2, decreasing mid-way through ~1800 cal by BP (Fig 4).

### Zone 3: 250 cal yr BP (= 1700 AD)– 2011 AD (63–0 cm)

**Fig 5:**
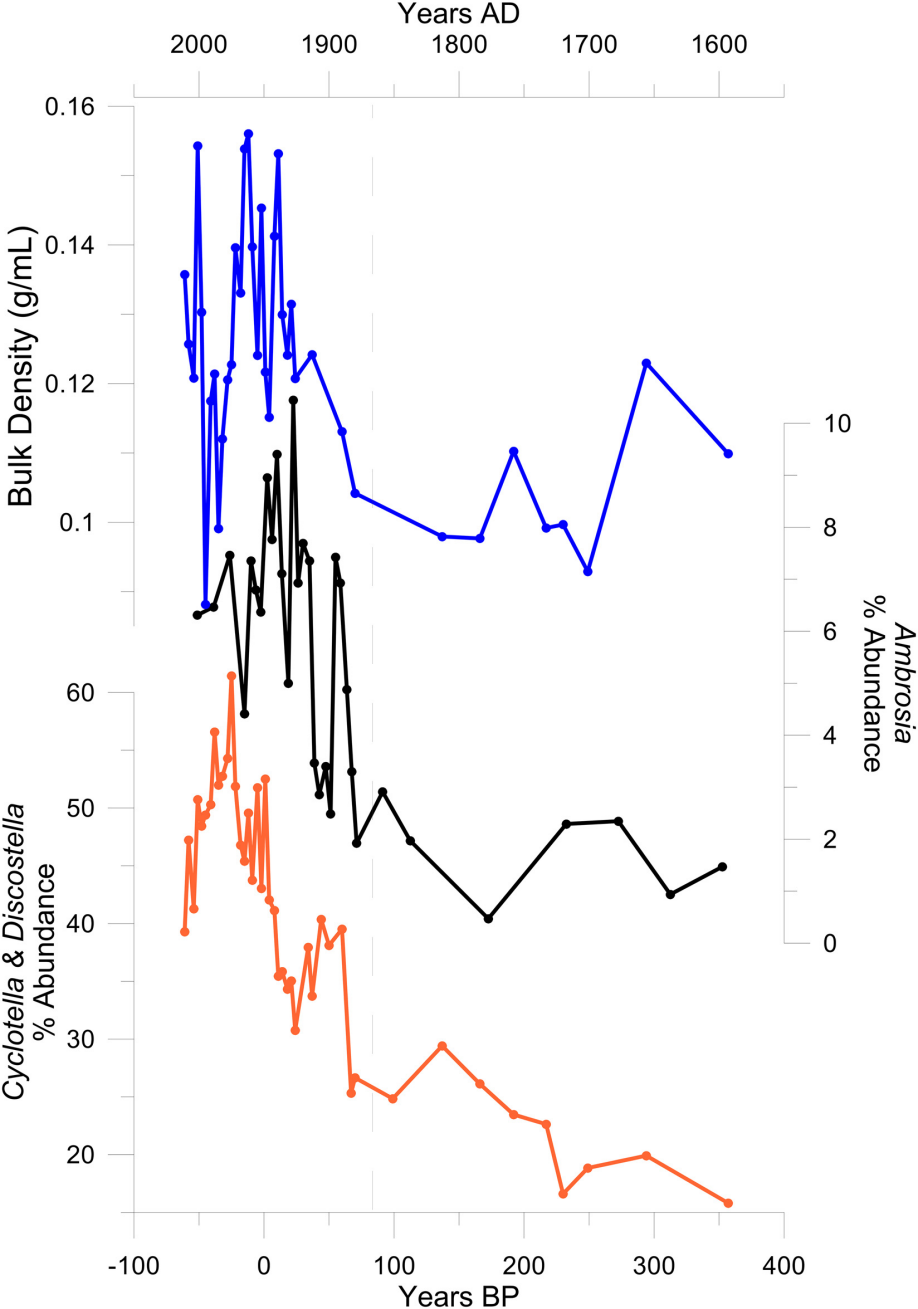
Post-settlement changes in Wishart Lake sediments. Changes in % abundance of *Cyclotella sensu lato* diatoms (orange curve), % *Ambrosia* pollen (black curve) and sediment bulk density (blue curve), are shown as a function of age in cal yr BP, with 0 yr BP = 1950 AD. Figure plotted using standard R plotting functions.

This zone is delimited on the basis of a further significant increase in *Lindavia bodanica* Nakov et al 2015, *L*. *Michiganiana* Nakov et al 2015, *Discostella pseudostelligera* and *D*. *stelligera* (Fig 3). The most significant increase in *Cyclotella sensu lato* spp. in this zone is temporally coincident with the *Ambrosia* rise (1880 AD); *Cyclotella sensu lato* spp. reach values >30%, which are unprecedented in the record. The samples dating to the most recent 30 years do not show any further increase in *Cyclotella sensu lato* spp., rather the abundances are stable or subtly declining (Fig 5). Cyst:diatom ratios in Zone 3 increase up to ~20% around 1900 AD then decline again following 1930 AD. Scale:diatom ratios are ~10%, with a sharp peak in a single sample to almost 40% ~1935 AD. LOI_550_ dips in Zone 3 between 1850–1920 AD, as LOI_950_ increases (Fig 3). The DCA axis scores show rapid and high magnitude change, indicating that the taxonomic shifts into Zone 3 are the most marked of the record (Fig 4).

### Loss-on-ignition

The mean value for the percent organic content of sediments as determined from percent mass lost at 550°C (LOI_55_) was 40% (± measurement error of 0.12%; Fig 3). There are significant increases in LOI_550_ from the base of the core to the surface, shown through a comparison of the LOI_550_ in the three diatom delineated zones using ANOVA (*p* < 0.05). There are two exceptions to this trend: declines in LOI_550_ between ~30–100 cal yr BP and ~6000–6500 cal yr BP (Fig 3). The LOI_950_ shows less change, with a Holocene average of 3.30% (± measurement error of 0.08%); the uppermost Zone 3 has significantly higher LOI_950_ (ANOVA, p < 0.05). The decreases in sediment organic matter (LOI_550_) and increases in inorganic carbon (LOI_950_) recorded in Zone 3 are also coincident with increases in sediment bulk density and the rise in *Ambrosia* pollen (Fig 5).

### Regional Pollen Record

Pollen data from nearby Upper Mallot Lake [20] were obtained for comparison between regional paleovegetation and changes in the aquatic environment at Wishart Lake as tracked by diatoms (Fig 4). At the inception of the Wishart Lake diatom record (7300 cal yr BP), the Upper Mallot pollen record indicates a mixed-forest assemblage dominated by *Pinus* spp, with *Alnus*, *Betula*, *Quercus* and *Ulmus* present as co-dominant or minor elements. A shift in pollen assemblages is noted around 4000 cal yr BP (onset of Zone 2c), with declines in pollen of *Pinus* and *Quercus* and concomitant increase in taxa indicative of more mesic conditions (*Acer saccharum*, *Betula*, Cupressaceae, *Picea*). Pollen-derived transfer functions reconstruct maximum Holocene July temperatures at 6000 cal yr BP, with a gradual decline to minima during the Little Ice Age ([20], and data publicly available in the Neotoma Database, http://www.neotomadb.org/]).

## Discussion

### Controls on Holocene aquatic ecosystem development

With a basal age of ~7300 cal yrs BP, the Wishart Lake sediment core did not capture the entire post-glacial record. Therefore, vegetation succession and the relationship to lake ontogeny immediately following deglaciation cannot be evaluated here. Nevertheless, the relatively high rate of sediment accumulation and robust age model permit analysis of mid- and late Holocene paleolimnology. Cluster analysis indicates significant shifts in diatom assemblages, driven mainly by fluctuations in the abundances of planktonic diatoms. A primary control on the abundance of planktonic diatoms is availability of open water habitat, determined in small, middle latitude lakes such as Wishart largely by water level. Thus, the changes in abundances of planktonic diatoms suggest that variability in lake water level through the pre-settlement part of the record is an important control on diatom assemblages.

The early portion of the Wishart Lake diatom record (Zone 1; Fig 3) is characterized by several indicators of lower water level, including lower abundances of planktonic diatoms, more benthic taxa, and a lower concentration of diatom valves. The base of the core consisted of compacted woody debris, supporting the interpretation of a low water stand around or just prior to 7300 cal yr BP. Benthic and epiphytic diatom taxa important in Zone 1 include *Achnanthidium minutissimum*, a broadly tolerant benthic taxon widespread in the study region, and *Brachysira brebissonii*, also a benthic taxon common in the study region, found preferentially in oligotrophic lakes with mildly acidic pH. *Pinnularia biceps* is also recorded in this zone, an epiphyte typically found in water with low mineral content [59]. *Aulacoseira distans* increases through Zone 1; analyses of lake surface samples across a transect of water depths in adjacent Michigan shows *A*. *distans* to be a benthic or tychoplanktonic species, present mainly in samples with water depths < 4 m [68]. Zone 1 assemblages as a whole indicate the availability of shallow water oligotrophic, circum-neutral to slightly acidic aquatic habitats.

Around 4000 cal yr BP, diatoms in the genera *Cyclotella sensu lato*, *Tabellaria* and *Aulacoseira* increase. These shifts delineate Zone 2, and suggest a possible increase in lake water level that persisted through the rest of the Holocene. Temporally coincident increases in lake level are recorded in paleolimnological studies to the west of the study site. *Cyclotella sensu lato* and other planktonic diatom taxa also increase after 4500 cal yr BP in three lakes in Ontario’s Experimental Lakes Area (ELA) [17, 69]. These increases were linked to rising water level (up to 8 m increase in Lake 239) through independent reconstructions of reduced effective evapotranspiration derived from pollen records from Lake 239 and a series of nearby lakes [16, 69]. While ELA sites are expected to show increased sensitivity to moisture availability because of their proximity to the prairie-boreal forest ecotone, our results confirm sensitivity of hemiboreal lakes several hundred km to the east of the prairie ecotone to shifts in moisture availability related to regional-scale Holocene climate fluctuations [70].

The diatom record from Wishart Lake suggests some role for water level changes in explaining long-term lake development at this site. However, given that the lake presently remains shallow and modern day water level variability is small, the record from Wishart Lake also suggests the possible influence of watershed processes on diatom assemblages. While the major shift in diatom community structure beginning around 4000 cal yr BP is marked by increases in *Discostella stelligera*, perhaps driven in part by regional climatically induced increases in water level, *Tabellaria flocculosa* also increases in Zone 2. *T*. *flocculosa* is generally interpreted as an indicator of nutrient enrichment [55, 71–74]. Lower cyst:diatom ratios and higher LOI_550_ in diatom Zone 2 further suggest the possibility of an increase in lake productivity, perhaps caused by a change in water quality along with water level rise after 4000 cal yr BP.

A possible driver of these changes in water quality relates to changes in paleovegetation. The pollen record from nearby Upper Mallot Lake shows a decline in *Pinus* pollen as *Betula* and other taxa more indicative of mesic conditions (*Acer saccharum*, *Picea*) increase, particularly after 4000 cal yr BP [20]. Pollen records from Jack Lake and Nina Lake in central Ontario, located in the same ecotonal region and at similar latitude to Wishart Lake, show a similar vegetation shift from *Pinus*-dominated coniferous forests to mixed hardwood forest, indicated by increases in pollen of *Betula* and other broad-leaved taxa, around 4000 cal yr BP [19]. This transition is also recorded at regional sites to the west including Lily Lake on Isle Royale, Michigan, a set of sites at Pictured Rocks National Lakeshore, Michigan [75], and Crooked Lake on the Upper Peninsula of Michigan [68]. These changes in paleovegetation are indicative of the well documented transition from warmer, drier middle Holocene climates to wetter, cooler late Holocene Neoglacial climates recorded locally at Upper Mallot Lake [20] and more broadly across Eastern and Mid-western North America [68, 76–78].

The regional increase in broad-leaved hardwood tree taxa after 4000 cal yr BP likely had important watershed biogeochemical effects. As needle-leaf litter is nutrient poor relative to litter produced by broad-leaved species [30], altered chemical composition of runoff would favour diatom assemblages more responsive to higher nutrient concentrations [79–82]. Multi-proxy studies of the effects on lakes of the middle Holocene decline in eastern North America of *Tsuga canadensis* document changes to lake nutrient concentrations in response to changes in leaf litter quality driven by the replacement of *Tsuga* with broad-leaved trees; the *Tsuga* decline often coincides with an increase in diatom taxa indicative of more eutrophic conditions [25, 26], including *Tabellaria flocculosa*. Thus, several processes, including enhanced nutrient flux due to runoff from a catchment increasingly vegetated with broad-leaved trees, and an increase in lake level resulting from a moister climate, may have combined to produce the observed shifts around ~4000 cal yr BP at Wishart Lake.

### Causes of recent changes

Zone 3 of the Wishart Lake diatom record contains assemblages unprecedented for the past 7300 years, suggesting critical effects of several facets of human activity on the system. Many Northern Hemisphere lakes show marked increases in *Cyclotella* spp. and declines in *Aulacoseira* spp. with climate warming, beginning in the late 19^th^ century for Arctic lakes, and the 1970s for temperate lakes [83]. In Wishart Lake, the increase in *Cyclotella* spp. is contemporaneous with the *Ambrosia* rise (Fig 5). This early date for the increase in *Cyclotella sensu lato* indicates a more complex response and allows for further discussion of the causes of the widespread rise in *Cyclotella sensu lato* diatoms in temperate lakes.

The *Ambrosia* rise is the result of multi-faceted landscape changes in the study region. Around 1880 AD mining became a dominant industry in nearby Sault Ste. Marie, Ontario. Many changes occurred such as land clearance, road and railway development, ore smelting and associated increases in acid rain and atmospheric deposition of nitrogen and other pollutants, and increased erosive fluxes into lakes [49, 50]. Therefore the correlation between the increase in *Cyclotella sensu lato* and the *Ambrosia* rise may not represent solely anthropogenic climate warming, particularly since syntheses of temperature records tracking anthropogenic warming do not show major temperature anomalies until into the 20^th^ century [84]. In a high resolution diatom record of the period following European settlement taken from a small lake proximal to Lake Michigan, Wolin and Stoermer [85] similarly show a distinct shift in assemblages in response to initial forest clearance, beginning around 1845 AD.

It has been suggested that recent increases in *Cyclotella sensu lato* may be related to enrichment by atmospheric deposition of nitrogenous compounds, as *Cyclotella sensu lato* abundance has been positively correlated in some studies with nutrient concentrations [6, 86, 87]. TLW has experienced minimal local changes in nutrient inputs due its status as a protected watershed with very limited settlement and it has not been extensively logged. While there has been an increase in regional atmospheric nitrogen deposition, there is no indication of nutrient enrichment at Wishart Lake based on an intensive monitoring program since the 1980s [42]. Thus, atmospheric deposition of N is unlikely to explain the recent increase in *Cyclotella sensu lato* at this site.

Increases in *Cyclotella sensu lato* occurring around the same time as the *Ambrosia* rise (1880 AD) with no strong evidence for acidification, large-scale atmospheric nutrient input or rapid climate change at that time, suggest that the observed diatom assemblage changes may reflect an effect of land clearance, vegetation change and associated changes in sediment fluxes to the lake, affecting water clarity. The relationship between the *Ambrosia* rise and *Cyclotella sensu lato* in watershed lakes is not well described however a correlation between them is recorded in a number of paleoecological studies of temperate lakes in North America [43, 68, 88, 89]. More high resolution multi-proxy records from temperate lakes have potential to provide new details on the autecology of the *Cyclotella sensu lato* group, and new insights into the complex mechanisms through which aquatic ecosystems are affected by human activity.

## Conclusion

Wishart Lake’s diatom community is largely driven by climate, and impacts of climate on water level, since 7300 cal yr BP. Vegetation succession driven by wetter and cooler conditions ~4000 cal yr BP, alongside potential rising lake levels caused significant increases in nutrient sensitive, planktonic taxa *Cyclotella sensu lato* spp. and *Tabellaria flocculosa*. Recent disturbance and landscape change associated with increased *Ambrosia* pollen correlates to unprecedented rises in *Cyclotella sensu lato* spp. This record shows Wishart Lake’s sensitivity to changes in the surrounding landscape caused by natural climatic variability pre-1880 AD, and by human impact thereafter. Further research in the Turkey Lakes Watershed is recommended to better understand the relationship between vegetation, aquatic communities and diatom assemblages in this changing region.
